# Hippocampal rejuvenation by a single intracerebral injection of one‐carbon metabolites in C57BL6 old wild‐type mice

**DOI:** 10.1111/acel.14365

**Published:** 2024-10-08

**Authors:** Alejandro Antón‐Fernández, Rocío Peinado Cauchola, Félix Hernández, Jesús Ávila

**Affiliations:** ^1^ Centro de Biología Molecular Severo Ochoa, CSIC‐UAM Madrid Spain; ^2^ Center for Networked Biomedical Research on Neurodegenerative Diseases (CIBERNED), Instituto de Salud Carlos III Madrid Spain; ^3^ Present address: Department of Neuroscience and Biomedical Sciences Carlos III University (UC3M) Madrid Spain

**Keywords:** adult neurogenesis, aging, cellular partial reprogramming, epigenetic clock, epigenetics, improved cognition, one‐carbon metabolites, transcription factors

## Abstract

The Izpisua‐Belmonte group identified a cocktail of metabolites that promote partial reprogramming in cultured muscle cells. We tested the effect of brain injection of these metabolites in the dentate gyrus of aged wild‐type mice. The dentate gyrus is a brain region essential for memory function and is extremely vulnerable to aging. A single injection of the cocktail containing four compounds (putrescine, glycine, methionine and threonine) partially reversed brain aging phenotypes and epigenetic alterations in age‐associated genes. Our analysis revealed three levels: chromatin methylation, RNA sequencing, and protein expression. Functional studies complemented the previous ones, showing cognitive improvement. In summary, we report the reversal of various age‐associated epigenetic changes, such as the transcription factor Zic4, and several changes related to cellular rejuvenation in the dentate gyrus (DG). These changes include increased expression of the Sox2 protein. Finally, the increases in the survival of newly generated neurons and the levels of the NMDA receptor subunit GluN2B were accompanied by improvements in both short‐term and long‐term memory performance. Based on these results, we propose the use of these metabolites to explore new strategies for the development of potential treatments for age‐related brain diseases.

## Graphical abstract

Reversal of Brain Aging Phenotypes. Microinjection of a cocktail containing four metabolites‐ putrescine, glycine, methionine and threonine (one‐carbon‐metabolite induction medium, 1C‐MIM) into the dentate gyrus of old mice results in the partial reversion of brain aging phenotypes. These changes include the reversion of the methylation patterns of specific age‐related chromatin components, RNA transcription changes, increased adult hippocampal neurogenesis, enhanced expression of young‐related synaptic proteins. Most importantly, this treatment also results in significant cognitive improvement.

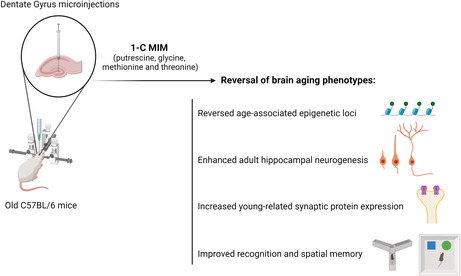

**Abbreviations**
1C: one carbon
1C‐MIM: one‐carbon metabolite induction medium
CNS: central nervous system
DEG: differentially expressed genes
DG: dentate gyrus
DNAm: DNA methylation
DNMT: DNA; methyltransferase
EWAS: epigenome wide association studies
FRα: folate receptor α
GCL: granular cell layer
MI: memory index
NMDA: N‐methyl‐D‐aspartic acid
NOR: novel object recognition
P‐H3: phospho histone H3
SAM: S‐adenosyl methionine
SGZ: subgranular zone
YF: yamanaka factors

## 1 INTRODUCTION

Aging is the major risk factor for several brain disorders, such as neurodegenerative diseases (Hou et al., 2019; Lopez‐Otin et al., 2013, 2023). It is strongly associated with the impairment of numerous cognitive functions, including episodic memory processing, which depends mainly on the hippocampus, including the dentate gyrus (DG) (Aimone et al., 2006; Hainmueller & Bartos, 2020). Furthermore, according to a growing number of studies, aging can be defined as a process driven by epigenetic alterations in chromatin components that render cells more dysfunctional, predisposing individuals to age‐related diseases (Horvath & Raj, 2018). Some of these alterations have been described as changes in the basal methylation state of some histone proteins or in DNA methylation (DNAm) patterns. These processes seem to be relevant during brain aging, for example, considering how DNAm regulates brain neuroplasticity, as well as learning and memory performance (Creighton et al., 2020; Levenson et al., 2006; Miller & Sweatt, 2007).

Fortunately, for potential anti‐aging treatment, both DNA and histone methylation have been described as reversible processes (Ramchandani et al., 1999; Shi et al., 2004), especially considering how mammals undergo natural dynamic variation throughout different stages of life (Hackett et al., 2012; Horvath & Raj, 2018). This reversibility can be artificially induced by Yamanaka factor (YF) overexpression both in vitro and in vivo (Planello et al., 2014; Wu et al., 2018). Furthermore, different in vivo genetic reprogramming strategies based on YF overexpression, which has been found to restore different age‐associated phenotypes, also reduce the epigenetic age of cells both in vitro and in vivo without loss of somatic identity (Lu et al., 2020; Ocampo et al., 2016; Olova et al., 2019). Partial reprogramming performed previously in our laboratory using old mice, based on cyclic induction of YF overexpression, prevented the age‐associated reduction in the histone 3 lysine 9 trimethylation epigenetic marker at the DG (Rodriguez‐Matellan et al., 2020). It was accompanied by the restoration of age‐associated phenotypes related to the neural central system, such as memory performance or neuroplasticity. Nevertheless, despite promising data from genetic models of partial reprogramming, the in vivo overexpression of YF is currently not a realistic strategy from a clinical point of view in humans. Consequently, in recent years, other more viable approaches have been taken into consideration in the search for easily delivered molecules able to restore epigenetic alterations in the brain to younger levels.

In this regard, compounds from one‐carbon (1C) metabolism could be an interesting option. 1C‐ metabolism is an ensemble of cyclic biochemical pathways that are involved in epigenetic regulation because they are regulators of histone and DNAm (Zeisel, 2009). Folate and methionine are the two main pathways involved in 1C metabolism, mediating the enhancement of DNA methyltransferases (DNMTs) or the synthesis of S‐adenosyl methionine (SAM), which is the major methyl donor in the cell for both DNA and histone methylation (Ducker & Rabinowitz, 2017; Lichtenwalner et al., 2001; Luo et al., 2013). Recently, Hernandez‐Benitez et al. reported that exposing cells to 1C metabolites favors plasticity traits (Hernandez‐Benitez et al., 2024). Specifically, they characterized a cocktail called 1C‐metabolite induction medium (1C‐MIM), which is able to promote partial reprogramming in cultured cells, including astrocytes, leading to accelerated muscle repair after injury in aged mice (Hernandez‐Benitez et al., 2024).

With the aim of exploring the in vivo effects of these reprogrammed compounds on the central nervous system (CNS) and to explore whether they can reverse brain aging phenotypes and reverse epigenetic alterations in age‐associated genes, we performed an intracerebral injection of several metabolites spatially restricted to the dorsal DG region in old wildtype mice. This area is a key region for memory performance (Hainmueller & Bartos, 2020), which is mainly affected by aging and by age‐related neurodegenerative diseases such as Alzheimer's disease (Drapeau et al., 2003; Geinisman et al., 1978; Moreno‐Jiménez et al., 2019; Ngwenya et al., 2015; Small, 2014). This is a novel in vivo pharmacological treatment performed on aged mice to induce rejuvenation of a brain region along with the reversion of different brain aging‐associated phenotypes, including important cognitive enhancement. In conclusion, the present study provides insight into the relationships among 1C metabolism, epigenetic mechanisms, transcription factors and aging within the brain.

## 2 RESULTS

### 2.1 Metabolite cocktail (1C‐4‐MIM) injected into the hippocampal‐DG area of aged C57BL6/J mice reversed age‐associated epigenetic changes

**FIGURE 1:**
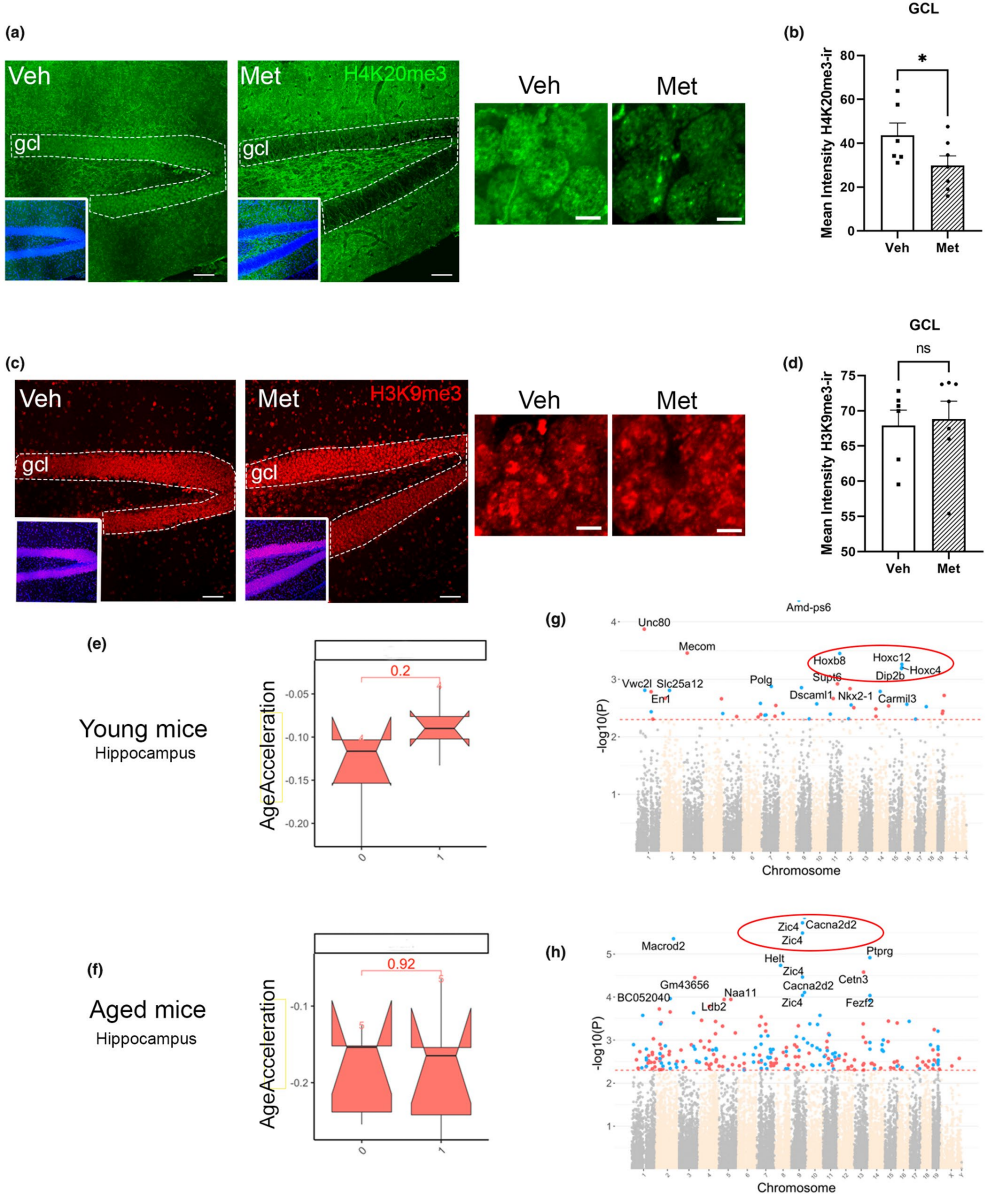
Epigenetic changes due to treatment with four compound cocktail metabolites. Age‐associated histone epigenetic markers: (a) Representative images of changes in H4K20me3 distribution in the dentate gyrus (DG) of 12‐month‐old wild‐type mice treated with vehicle (Veh) or metabolites (Met). On the right, a high‐magnification image of nuclei expressing the H4K20me3 marker is shown. (b) Levels of H4K20me3 were obtained by determining the mean intensity of immunoreactivity in the DG. Treatment with metabolites induced a significant reduction in H4K20me3 expression in the DG. (c) Representative images of H3K9me3 distribution in the dentate gyrus (DG) of 12‐month‐old wild‐type mice treated with vehicle (Veh) or metabolites (Met). On the right, a high‐magnification image of nuclei expressing the H3K9me3 marker is shown. (d) Levels of H3K9me3 were obtained to determine the mean intensity of immunoreactivity in the DG. Treatment with metabolites did not significantly change H3K9me3 expression in the DG. Mean ± SEM; *p < 0.05, Student's *t* test; n.s., no significant difference. The black square represents mice infused with vehicle; the black dots represent mice infused with metabolites. DNA methylation levels of age‐associated loci: (e, f) Horvath epigenetic clock analysis did not reveal a significant reduction in hippocampal epigenetic age in mice treated with one‐dose metabolite injection (Met) either at 4 months of age (e) or at 16 months of age (f). (g, h) Manhattan plots of DNAm aging loci in young (g) and aged (h) mice affected by metabolite treatment, representing the loci with a divergent DNAm aging pattern between control and treated mice. The coordinates are estimated based on the alignment of the *Mus musculus* array probes. The direction of associations with *p* < 10–3 (red dotted line) is highlighted by red (hypermethylated) and blue (hypomethylated). The top 15 CpGs were labeled according to their neighboring genes.

It has been shown that certain epigenetic modifications involved in the maintenance of heterochromatin are differentially regulated during aging. Two key epigenetic marks associated with aging, H3K9me3 and H4K20me3, change with age (Benayoun et al., 2015; Liu, Cao, et al., 2013). Trimethylation of the two main nucleosome histones H3 and H4 has been related to the repression of gene expression in heterochromatin regions present in young mice (Padeken et al., 2022). To test whether treatment with four compound cocktail metabolites (1C‐4‐MIM, see Methods) in the hippocampus could lead to changes in histone methylation of both epigenetic marks to younger‐like levels, we examined the immunofluorescence expression of H4K20me3 and H3K9me3 (Figure 1a–d) in the granular cells of the DG from 12‐month‐old mice. The immunoreactivity of H4K20me3 in the granular layer, which is basically composed of granular neurons, was significantly different between control and 1C‐4‐MIN‐treated mice (Figure 1b). Figure 1b shows that a simple dose of metabolite injection reduces H4K20me3 epigenetic marker levels. H4K20me3 is a marker that increases in liver and kidney of aged rats, as well as in human fibroblasts (Benayoun et al., 2015). However, treatment of 12‐month‐old mice was not sufficient to induce changes in the levels of H3K9me3 (Figure 1d), a marker that decreases with age (Benayoun et al., 2015).

There is a relationship between histone methylation and DNAm (Henckel et al., 2009), which work together in gene silencing, as in the case of cellular reprogramming (Mikkelsen et al., 2008). Thus, we explored potential changes in other epigenetic marks, such as DNAm. A few years ago, Horvath developed the first multitissue age estimator (Horvath's clock) able to measure age by analyzing DNAm from 353 different CpGs of different tissues (Horvath, 2013). Although many of these CpG methylations show only a weak correlation with chronological age, the analysis of the whole methylation pattern emerges as an effective biomarker of aging based on DNAm clock data. To date, Horvath's clock has been shown to be accurately correlated with chronological age (Horvath, 2013), and it also works well for the hippocampus (Coninx et al., 2020). Thus, our next step was to test Horvath's epigenetic clock in the hippocampus of our C57BL6/J mice at two different ages. For this purpose, we analyzed hippocampal CpG methylation in young (~4 months old) and aged (~16 months old) mice injected with either vehicle or metabolites. Horvath's methylation clock analysis revealed, for the first time, that the hippocampi of 4‐ and 16‐month‐old vehicle‐treated mice exhibited slight differences in chronological age. Both showed very similar values of age acceleration that were practically equal to zero (−0.12 and − 0.15, respectively) (Figure 1e,f). Treatment with 1C metabolites had a similar effect. Thus, when Horvath's clock method was applied to the hippocampus, we did not observe significant changes in the rate of age acceleration due to single‐dose metabolite treatment either at 4 months of age (−0.09) or at 16 months of age (−0.16). However, although the epigenetic clock used for this analysis was designed to correlate the methylation levels of several CpGs, these were only a small fraction of all CpGs related to age‐associated changes according to epigenome‐wide association studies (EWAS). By studying all the CpGs associated with aging and setting a p value <10–3 as the cutoff for significance (Figure 1g,h), we found that in aged mice, metabolite treatment reversed the methylation levels of 248 loci (Figure 1h). Overall, 143 (58%) corresponded to hypermethylation, and the remaining 105 (42%) corresponded to hypomethylation.

The two top age‐associated genes (highlighted in red in Figure 1h) that underwent the most significant changes related to metabolite effects (*p* <10–5, FDR <0.05) were Zic4 (four different sequences detected), which encodes a transcription factor expressed in the mouse nervous system that has been recently associated with hippocampal aging and spatial memory (Chiavellini et al., 2022), and Cacna2d2 (two different sequences detected), which encodes a subunit of voltage‐dependent calcium channels associated with synaptic plasticity. In addition, we detected changes in other top age‐associated genes (Figure 1h) significantly affected by metabolites (*p* <10–4): Macrod2 (encoding an ADPr monohydrolase highly expressed in neurons and associated with behavior), Ptprg (encoding a receptor protein tyrosine phosphatase associated with memory performance and downregulated with age), Helt (encoding a transcription factor required for the development of interneurons), Cetn3 (encoding a calcium‐binding phosphoprotein with an important role in cell division and neurodevelopment), Fezf2 (encoding a relevant developmental transcription factor expressed in glia in the DG and essential for neuronal differentiation), GM43656 (unknown function), Naa11 (N‐alpha‐acetyl transferase), BC052040 or Cdin1 (encoding a nuclease associated with neuronal differentiation), and Ldb2 (encoding a transcription factor involved in somatic stem cell population maintenance, nervous system development and modulation of synaptic function). All these changes were detected in 16‐month‐old mice, 7 of which exhibited hypomethylation (Zic4, Cacna2d2, Macrod2, Ptprg, Helt, Fezf2, and Cdin1), and 4 of which exhibited hypermethylation (Cetn3, Ldb2, Naa11, and GM43656).

It is important to note that the epigenetic changes caused by 1C metabolite treatment in younger mice were five times less effective than those in older mice, where we detected changes only in 44 CpGs associated with age (Figure 1g). In addition, the proportion of hypo−/hypermethylated CpGs was much greater than that in older mice, with 24 hypomethylation events and 21 hypermethylation events.

Among the 44 CpGs in younger mice (~4 months old), the age‐associated genes that underwent the most significant changes due to metabolite effects (*p* <10–5, FDR <0.05) were related to 1C metabolism, such as Amd‐ps6 (S‐adenosylmethionine decarboxylase) or Dip2b (encoding a protein that contains a binding site for DNA methyltransferase 1 and is near a fragile folate‐sensitive site); early development, such as the Mecom or homeobox genes Hoxb8, Hoxc4 or Hoxc12; and brain disorders, such as Slc25a12 (which is present at high levels in parvalbumin interneurons), Polg or Unc80 genes.

### 2.2 Effect of single‐dose injection of metabolites on the transcriptome (RNA‐seq)

**FIGURE 2:**
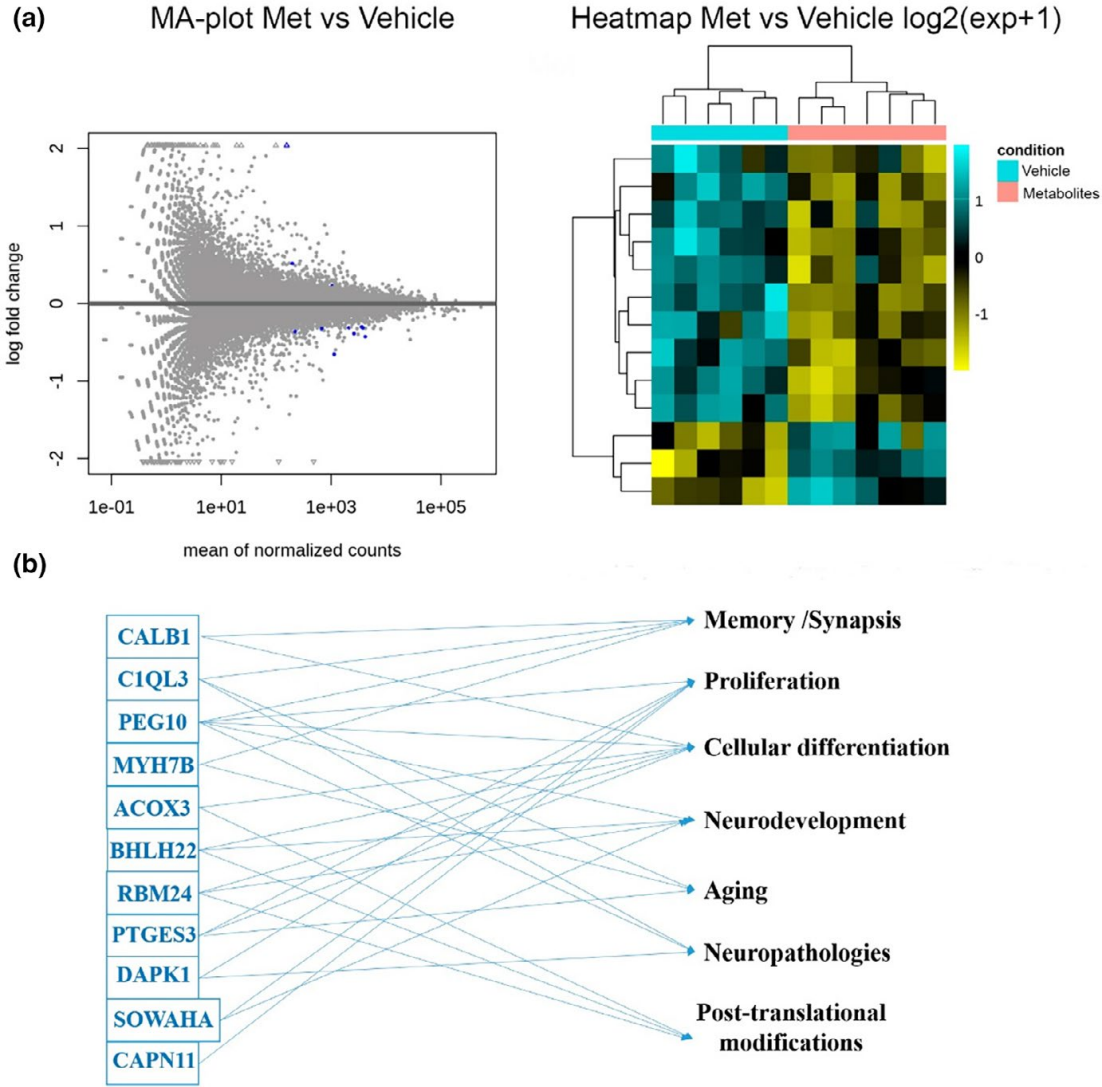
Effect of metabolite treatment on differential gene expression according to RNA‐seq data. (a) Genes colored in blue have *p* values less than 0.05; points that fall out of the window are plotted as open triangles pointing either up or down. The heatmap is shown on the right, and the color and intensity in the heatmap represent changes in gene expression from the list of genes with q‐values. (b) Roles in which different genes were up‐ or downregulated by treatment.

Changes in the methylation of chromatin components could result in changes in RNA transcription. Thus, we studied whether metabolite treatment in the aged hippocampus could result in changes in the expression of RNAs encoding proteins expressed at younger ages. A summary of the RNA‐seq analysis (ENA accession number: “PRJEB44560”, and the unique name was “ena‐STUDY‐CBMSO‐27‐04‐2021‐12:23:29:210–1248”), with the number of differentially expressed genes (DEGs) (*q* <0.05) in the presence of metabolites, is shown in Table 1. The gene expression data were graphically summarized with different types of graphics. The MA plot shows the log2‐fold changes (M) between two conditions over the mean of normalized counts (A) for all samples (Figure 2a). In the heatmap (Figure 2a), the data are displayed in a grid where each row corresponds to a gene and each column corresponds to a sample (from two different conditions). The RNA‐seq results showed that single‐dose metabolite treatment induced significant, albeit modest, changes in the transcriptome of a reduced group of genes that are implicated in common relevant functional features (see Table 1; Figure 2b). Notably, except for Zfp968‐ps and AC115970.1, which have no known functions or are reported in the literature, all of these genes are involved in proliferation, cellular differentiation and neurodevelopment. This may imply that partial reprogramming at the DG may be ongoing due to metabolite treatment. For example, seven of thirteen genes detected by RNA‐seq (Acox3, BHLHE22, DAPK1, CALB1, PTGES3, PEG10, and RBM24) have been directly implicated in neuronal differentiation in young mice; also, seven of 13 genes (CAPN11, BHLHE222, DAPK1, C1qL3, PTGES3, PEG10, and SOWAHA) have a role in cellular proliferation (Akamatsu et al., 2015; Qin et al., 2014; Wong et al., 2016; Zhong et al., 2021). Notably, six of those 13 genes (MYH7B, DAPK1, CALB1, C1qL3, PTGES3, and PEG10) have been shown to be relevant to memory function (Dumas et al., 2004; Guo et al., 2019; Martinelli et al., 2016; Minhas et al., 2021; Rubio et al., 2011). Some of these genes are also involved in aging, and prostaglandin E synthase (PTGES3), an enzyme involved in prostaglandin‐2 synthesis, has been shown to be upregulated in aging‐induced cognitive decline (Minhas et al., 2021).

**TABLE 1:** Names of differentially expressed genes (DEGs) in the hippocampus according to vehicle treatment and their reported functions.

DEG	Expression value	Function
CAPN11	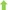 2098	Proliferation, differentiation, extracellular matrix
MYH7B	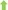 0,516	Memory, synaptic regulation, aging
ACOX3	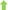 0,227	Differentiation, epigenetic regulation, neurodevelopment
DEG	Expression value	Function
PEG10	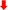 −0,655	Neurodevelopment, proliferation, differentiation, Memory
CALB1	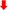 −0,428	Neuroplasticity, Memory, neuronal differentiation
C1QL3	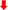 −0,388	Aging, granular cells differentiation, synaptic regulation, proliferation, Extracellular matrix
Zfp968‐ps	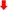 −0,361	Unknown
RBM24	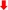 −0,321	Differentiation stem cell, neurodevelopment
AC115970.1	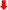 −0,315	Unknown
BHLHE22	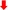 −0,313	Hippocampal neurodevelopment, neuronal differentiation, transcription factor
SOWAHA	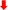 −0,302	Proliferation, neurodevelopment
PTGES3	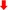 −0,231	Aging, neuronal proliferation and differentiation, memory, neuroprotection
DAPK1	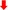 −0,154	Proliferation, neurodevelopment, neuroprotection, neuronal differentiation, memory

It should be taken into account the possible pleiotropic effects of some of those metabolites. For example, methionine and glycine are glutathione precursors and, in the case of methionine, it is via the trans‐sulphuration pathway, yielding cysteine. It may change cell redox status protecting against oxidative insults.

Thus, we have looked at the possible correlation between the observed changes related to the presence of 1C metabolites and the expression of any of the genes indicated in Figure 2 that could be related to redox status. In this way, it was reported that DAPK1 expression and activity could be affected by the redox status of the cells (DeGregorio‐Rocasolano et al., 2020). This possible side effect of methionine will require further analysis.

### 2.3 Effects of metabolites on the expression of factors involved in reprogramming

Previous results from Hernandez‐Benitez et al. showed how 1C‐MIM metabolites induced intermediate cell states in cells such as cultured astrocytes (Hernandez‐Benitez et al., 2024).

**FIGURE 3:**
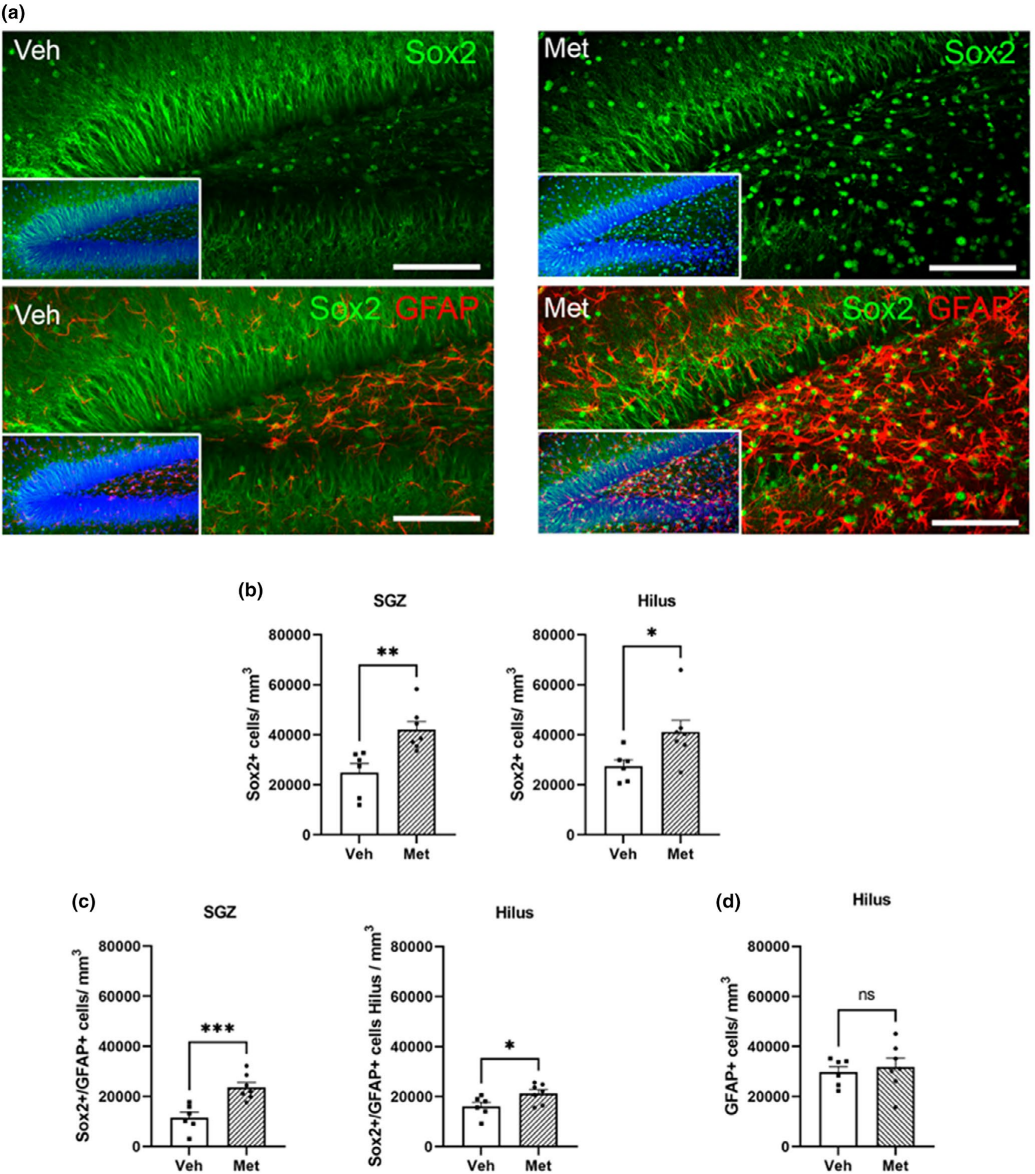
Effect of metabolites on Yamanaka factor Sox2 protein expression and in radial glial‐like stem cell population. (a) Representative confocal images showing changes in the number of Sox+ cells in the dentate gyrus (DG) of 12‐month‐old wild‐type mice treated with vehicle (Veh) or metabolites (Met). Dapi, blue; Sox2, green; GFAP, red. (b) Density of Sox2+ cells per mm^3^ obtained in the subgranular zone (SGZ) and in the hilus. (c) Density of Sox2+/GFAP+ cells per mm^3^ obtained in the subgranular zone (SGZ) and in the hilus. (d) Density of GFAP+ cells per mm^3^ obtained from the hilus. Treatment with metabolites induced a significant increase in the number of Sox2+ and Sox2+/GFAP+ cells in both regions of the DG (mean ± SEM; *p < 0.05, **p < 0.01, ***p < 0.001; Student's *t*‐test) but not in the case of the GFAP+ cell population. The black square represents mice infused with vehicle; the black dots represent mice infused with metabolites.

After the RNA‐seq results from our injected mice revealed potential reprogramming effects due to metabolite treatment on the DG and because of the Sox2 hypomethylation induced by metabolites, we analyzed changes in the presence of the Sox2 protein, one of the four YF whose expression is essential for cellular reprogramming to younger states. We analyzed mainly the subgranular zone (SGZ) and hilus, where we expected the majority of effects to be due to the stereotaxic injection of metabolites (Figure 3a). We detected a significant increase in Sox2+ brain cells from both hippocampal regions in metabolite‐treated mice (Figure 3b). The overall increase in the Sox2 population suggested that metabolite treatment induced partial reprogramming similar to that reported by Hernandez‐Benitez et al. in muscle cells (Hernandez‐Benitez et al., 2024). The mechanism for this So ×2 increase could be related to that found recently (Anton‐Fernandez et al., 2024). In that study, it was shown that other small compounds, such as folate, could bind to folate receptor alpha (FRα), a protein mainly present in neuronal cells (The Human Protein Atlas, n.d.), in the first step of partial reprogramming. The binding of folate to FRα results in the transport of FRα to the cell nucleus, where it can act as a transcription factor, facilitating the expression of YF (Anton‐Fernandez, Cuadros, et al., 2024); see also (Mohanty et al., 2016).

Thus, we tested whether the metabolite cocktail facilitates nuclear FRα transport (Figure S1a). Our results indicated that the metabolite cocktail facilitates the transport of FRα to the nucleus (Figure S1b) when it is added to a culture of SK‐N‐SH cells. Thus, such cocktails behave like folate or related compounds (Anton‐Fernandez, Cuadros, et al., 2024). Additionally, an increase in Kfl4 expression was also detected in the presence of the metabolites (Figure S1c,d).

Additionally, we detected an increase in the number of Sox2+/GFAP+ cells (Figure 3c), as both Sox2 and GFAP are markers of radial glia‐like cells. However, no changes in GFAP+ cell density were found (Figure 3d). The overall increase in Sox2 expression and the Sox2/GFAP+ cell population suggest that metabolite treatment induces in vivo partial reprogramming similar to that observed by Hernandez‐Benitez et al. (Hernandez‐Benitez et al., 2024). Considering the key role of radial glial‐like stem cells in adult neurogenesis, we next studied the effects of metabolites on this issue.

### 2.4 Effects of metabolite injection into the DG on cell proliferation

**FIGURE 4:**
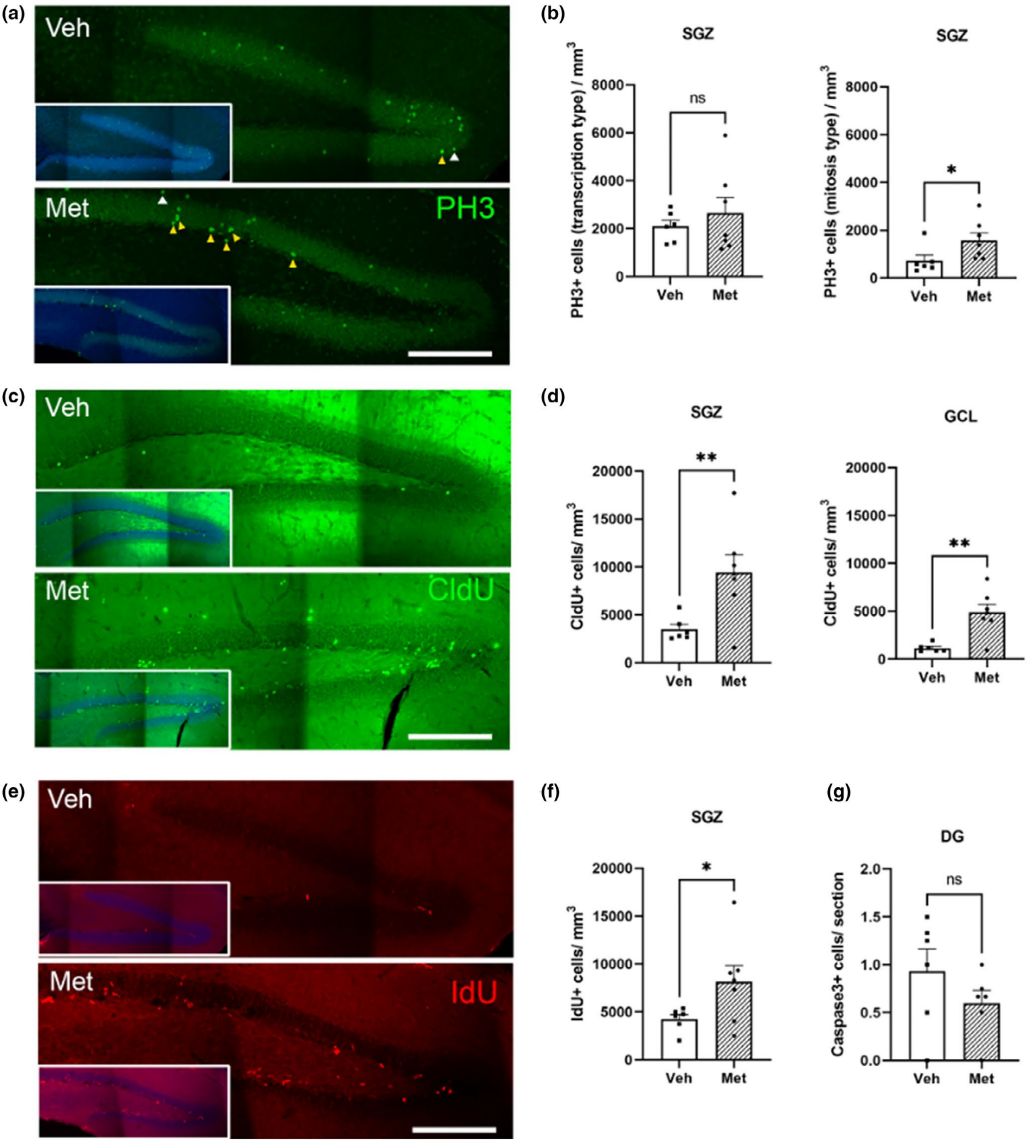
Effect of metabolites on proliferation and apoptosis. (a) Representative stitched confocal images of phospho‐histone H3 (PH3) (Ser10) “transcription type” (white arrowheads) and “condensation type” (yellow arrowheads) cells in the dentate gyrus (DG) of 12‐month‐old wild‐type mice treated with vehicle (Veh) or metabolites (Met). DAPI is blue; PH3 is green. All “condensation type” cells found in those representative images are indicated in yellow. (b) Density of PH3+ “transcription type” cells per mm^3^ obtained in the whole granular cell layer and PH3+ “condensation type” cells in the subgranular zone (SGZ). Treatment with metabolites induced a significant increase in cell proliferation in the SGZ. (c) Representative stitched confocal images of changes in the number of 2‐week‐old CldU+ cells in the dentate gyrus (DG) of 12‐month‐old wild‐type mice treated with vehicle (Veh) or metabolites (Met). Dapi is blue; CldU is green. (d) Density of CldU+ cells per mm^3^ obtained in the subgranular zone (SGZ) and in the granular cell layer (GCL). Treatment with small compounds induced a significant increase in the number of CldU+ cells in the DG. (e) Representative stitched confocal images of changes in the number of IdU+ 24‐hour‐old cells (white arrowheads) in the DG of 12‐month‐old WT mice treated with vehicle (Veh) or metabolites (Met). Dapi is blue; ldU is red. (f) Density of ldU+ cells per mm^3^ obtained in the subgranular zone (SGZ). Treatment with small compounds significantly increased the number of ldU+ cells in the DG. (g) Density of Caspase3+ cells per mm^3^ obtained in the subgranular zone (SGZ). Mice treated with metabolites did not appear to exhibit any increase in apoptotic cell death. As shown in all histograms, the black squares represent mice infused with vehicle, and the black dots represent mice infused with metabolites. All the data are expressed as the means ± SEMs and were analyzed by Student's *t*‐tests; *p < 0.05, **p < 0.01, n.s. no significant differences.

It has been previously shown that reprogramming induced by YF increases proliferation (Wang et al., 2019). On the basis of our RNA‐seq results showing that the expression of genes involved in proliferation, such as the increase in calpain and Sox2 protein expression, increased, we decided to study the effect of metabolite treatment on proliferative immunofluorescent markers. Different antibodies targeting phospho‐histone H3 (P‐H3) and two different thymidine analogs were used. Immunofluorescence analysis of P‐H3 revealed two types of staining, one of which was darker than the other (Figure 4a). These staining types have been reported previously in hippocampal neurons (Crosio et al., 2003; Rodriguez‐Matellan et al., 2020) and other cells (Cheung et al., 2003). Darker staining (condensation) (see the yellow arrow in Figure 4a) indicates an increase in chromosome condensation during mitosis and may indicate a greater degree of cell proliferation (Crosio et al., 2003; Goto et al., 1999). In contrast, the lighter staining may be related to gene expression (transcription) at interphase (Chadee et al., 1999; Mahadevan et al., 1991) (see the white arrow in Figure 4a). Differences between control and treated mice in the level of P‐H3 related to the “chromatin condensation type” (mitosis) were observed in the SGZ but not in the “chromatin transcription type” (Figure 4b), suggesting an increase in proliferation in the adult neurogenesis region. After the intraperitoneal (IP) administration of CldU 24 h before metabolite injection (Figure 4c,d) or of IdU 24 h before mouse sacrifice (Figure 4e,f), we were able to study the effect of the metabolite treatment on new cell survival and proliferation, respectively. Our results revealed a significant improvement in the survival of newly generated cells, as indicated by an increase in the number of CldU+ cells in both the SGZ and granular cell layer (GCL) (Figure 4d). This increase in both areas indicates that metabolite treatment induced a better survival rate for newly generated cells, which were able not only to survive at SGZ but also to integrate GCL circuits. The increase in IdU+ cells due to treatment confirmed the positive effect of metabolites on cellular proliferation in this neurogenic niche (Figure 4f). Interestingly, none of these alterations resulted in a decrease in the number of dead cells, as the number of caspase‐3+ cells did not increase (Figure 4g).

### 2.5 Impact of metabolite treatment on adult neurogenesis in the DG

**FIGURE 5:**
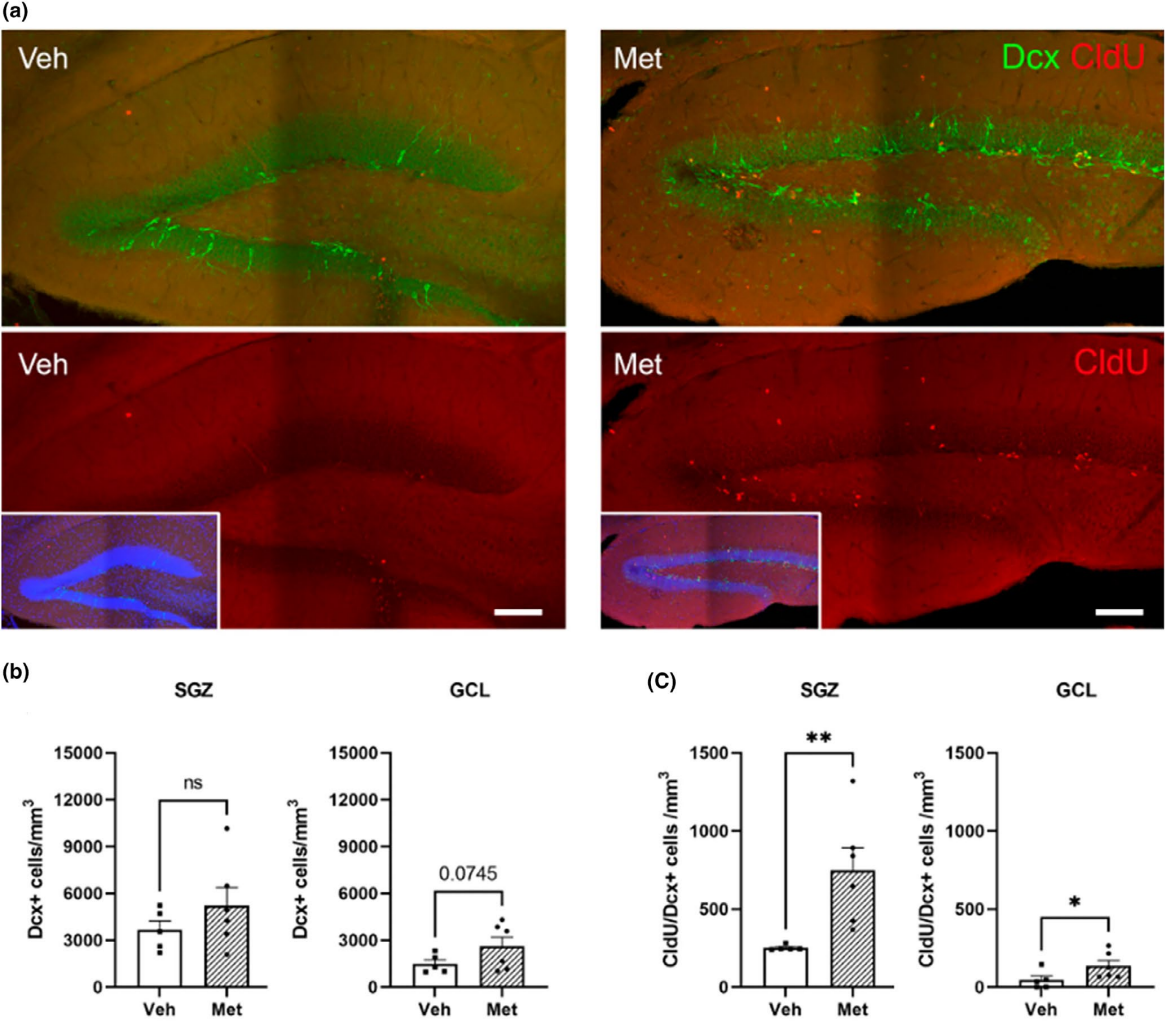
Effect of metabolites on adult neurogenesis. (a) Representative stitched confocal images of double‐labeling immunofluorescence for Dcx (green) and CldU (red) in the dentate gyrus of 12‐month‐old wild‐type mice treated with vehicle (Veh) or metabolites (Met). DAPI is shown in blue. (b) Density of Dcx + cells per mm^3^ obtained in the subgranular zone (SGZ) and in the granular cell layer (GCL). (c) Density of double‐labeled Dcx/CldU+ cells per mm^3^ in the subgranular zone (SGZ) and in the granular cell layer (GCL). Treatment with metabolites tended to increase the number of doublecortin+ cells in the granule cell layer (*p* = 0.0745) and significantly increase the density of Dcx/CldU+ cells in the dentate gyrus (mean ± SEM; *p < 0.05, **p < 0.01; Student's *t*‐test). For all histograms, black squares represent mice infused with vehicle; black dots represent mice infused with metabolite treatment.

The specific increase in cell proliferation and Sox2/GFAP+ radial glial‐like stem cells in the neurogenic niche of the SGZ following metabolite treatment, along with the increase in factors related to neuronal differentiation, led us to study the effect of these metabolites on neurogenesis. Thus, we used doublecortin antibodies to analyze the effect of metabolites on newly generated neurons (Figure 5a). In addition to a strong tendency toward increased density (particularly in the GCL) of doublecortin‐positive cells (Figure 5b), we observed a significant increase in the number of double‐labeled DCX+/CldU+ cells in the SGZ (Figure 5c). These results indicated that the administration of metabolites to the DG led to an increase in adult neurogenesis, mainly by improving the survival ratio of newly generated young neurons, which has been shown to be impaired by aging.

In summary, after metabolite treatment, mice showed increased proliferation and survival of new neurons generated in the SGZ of the DG, reverting important age‐associated phenotypes to younger levels.

Considering the increase in the number of new neurons in the DG, along with previous epigenetic and RNA‐seq results indicating potential improvements in synaptic function and cognition, we sought direct evidence of increased neuroplasticity.

### 2.6 Metabolites increase GluN2B subunit receptor levels but not GluR1 expression

It has been previously shown that the overexpression of YF in the hippocampus induces the expression of proteins such as GluN2B, which is expressed specifically in young animals (Rodriguez‐Matellan et al., 2020). The hypomethylation of genes encoding channel subunit receptors involved in regulating synaptic plasticity, such as Cacna2d2, and the results from RNA‐seq revealing increased expression of genes involved in synaptic regulation led us to evaluate the DG for potential improvements in neuroplasticity. We used a key neuroplasticity marker, Glun2B, whose expression decreases with age (Paoletti et al., 2013; Pegasiou et al., 2020; Shipton & Paulsen, 2014), along with another subunit expressed mainly in mature cells, the α‐amino‐3‐hydroxy‐5‐methyl‐4‐isoxazole propionate (AMPA) receptor subunit GluR1 (Hagihara et al., 2011).

**FIGURE 6:**
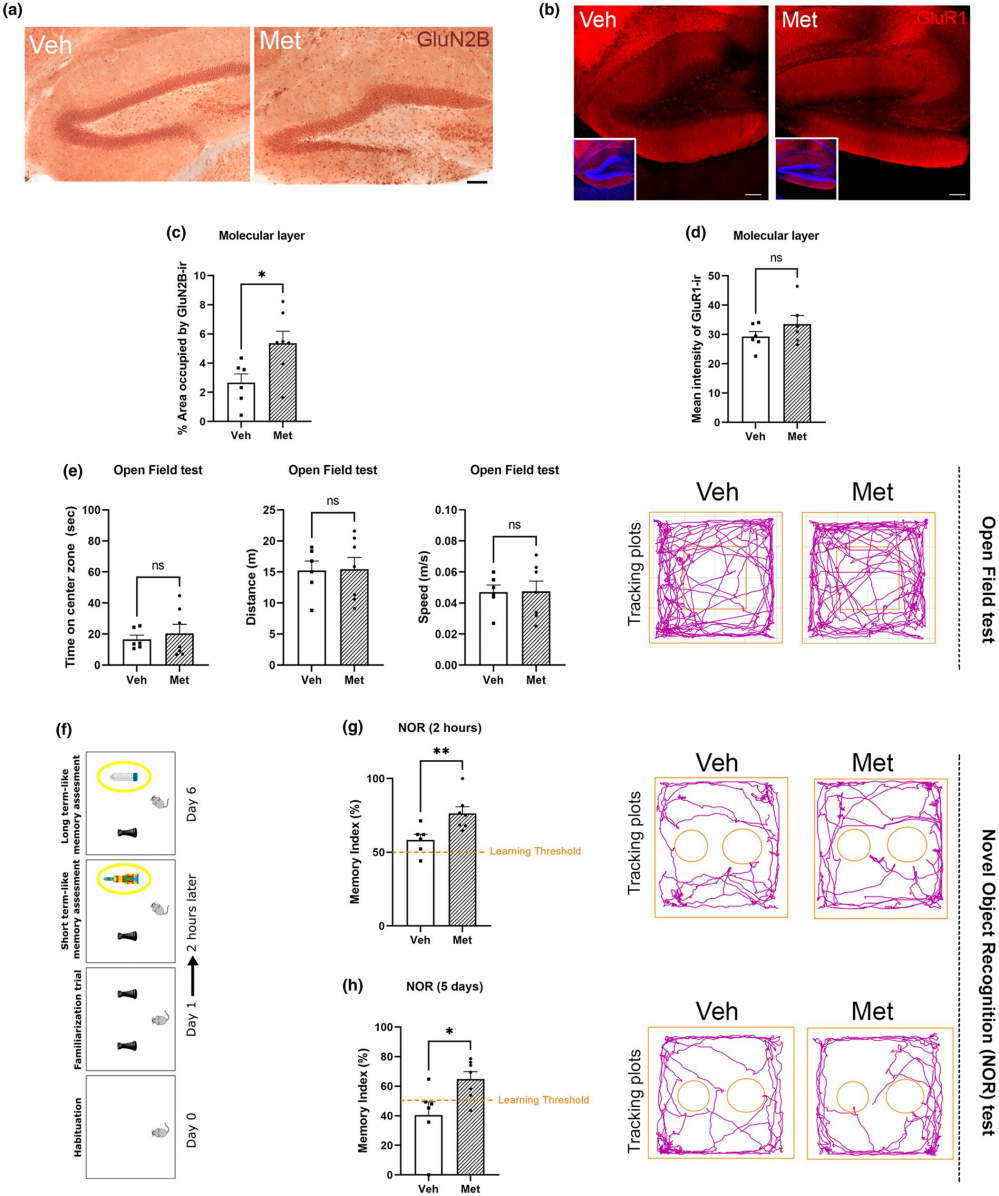
Effect of metabolite treatment on age‐related synaptic receptor subunits and on behavior and cognition. (a) Representative immunohistochemical images showing the distribution of GluN2B in the dentate gyrus (DG) of 12‐month‐old wild‐type mice treated with vehicle (Veh) or metabolites (Met). (b) GluR1 levels were obtained by determining the percentage of area occupied by the marker on the DG. Treatment with metabolites induced a significant increase in GluN2B expression in the DG (mean ± SEM; **p < 0.01, Student's *t*‐test). (c) Representative immunohistochemical images showing the distribution of GluN2B in the dentate gyrus (DG) of 12‐month‐old wild‐type mice treated with vehicle (Veh) or metabolites (Met). (d) The levels of GluR1 were obtained by determining the mean intensity of immunoreactivity in the DG. Treatment with metabolites did not induce a significant increase in glutamate receptor expression in the DG (mean ± SEM; Student's *t*‐test). For all histograms, black squares represent mice infused with vehicle; black dots represent mice infused with metabolite treatment. (e) Histograms show some of the main data from the open field test: Time spent by mice in the center square of the box, total distance traveled and average speed of movement. metabolites‐treated mice have not showed changes in any parameter studied: Anxiety‐depression‐like behavior (indicated by the time spent in the center square), as well as in general motor activity (illustrated by the rest of parameters). On the right are displayed tracking plots depicting two representative examples from each experimental group. (f) Diagram illustrating the different stages of the NOR test procedure. The day after habituation, the mouse was allowed to explore two identical replicates of an object (rooks), for 5 min. After 2 h a novel object (indicated by yellow circles) was introduced and the animal was allowed to explore the familiar and the novel object for 5 min. A new object was introduced 5 days later in this test phase, during the same time as before. (g) The memory performance analyzed 2 h after the familiarization trial showed that metabolites‐treated mice performed significantly better than control group. In orange is showed the learning threshold (50%), below it theoretically the mouse has not learnt. On the right are displayed tracking plots depicting two representative examples from each experimental group. (h) After 5 days, wild‐type mice treated with metabolites showed a significant memory retention, while almost all control mice had forgotten the object included in the previous test. On the right are displayed tracking plots depicting two representative examples from each experimental group. For all histograms, black square represents mice infused with vehicle; black dots represent mice infused with metabolites treatment. All data were expressed as mean ± SEM and analyzed by Student's *t*‐tests. *p < 0.05, **p < 0.01.

It is present in the N‐methyl‐D‐aspartic acid (NMDA) receptor, facilitating calcium permeability and increasing synaptic potentiation (Tang et al., 1999; Yashiro & Philpot, 2008). GluN2B immunoreactivity in the DG, particularly in the molecular layer, was greater in metabolite‐treated mice than in vehicle‐treated mice (Figure 6a,b), while GluR1 expression did not significantly change (Figure 6c,d). The observed increase in GluN2B reflects changes in neuronal plasticity, reverting to an age‐associated phenotype characterized by a progressive decrease. This neuroplasticity enhancement could lead to better cognitive functioning, and for that reason, we checked the potential enhancement of memory by testing memory performance tests such as the NOR test.

### 2.7 Enhanced memory performance assessed through behavioral tests

#### 2.7.1 Improvement of memory performance using the object recognition test

Prior to assessing cognitive performance in these mice, we examined potential behavioral alterations resulting from metabolite administration using the open field test. The results did not reveal changes in locomotor activity (distance or speed) or anxiety‐related behavior (time spent in the center area) due to metabolite treatment (Figure 6e).

It is well known the aging dependent loss of late memory in old mice compared with young mice, see for example (Wimmer et al., 2012). In this way, to study potential cognitive enhancement due to metabolite treatment, we used the NOR test (Figure 6f). Two hours after object familiarization, all mice were able to recognize the new object with a memory index (MI) above the learning threshold, but those treated with metabolites performed significantly better than control mice (Figure 6g). This memory improvement in the treated mice was maintained at 5 days postfamiliarization (Figure 6h), and the mice properly recognized a new second object.

#### 2.7.2 Improvement of memory performance using Y‐maze spatial memory test

Moreover, to compare cognition in young and old mice to look further for the effect of 1C metabolites in old mice, spatial memory was analyzed using the Y‐maze. This experiment also included a group of young mice injected with either the vehicle or the metabolites. The results showed a significant improvement in spatial memory in the aged mice injected with the metabolites, bringing their MI to levels comparable to those of the young mice injected with the vehicle (Figure S2).

In summary, treatment with metabolites had a positive effect on memory performance, both in the short and long term.

## 3 DISCUSSION

In the present study, we addressed some relevant age‐dependent alterations found in brain areas susceptible to premature degeneration related to aging, such as the DG. It has been suggested that brain structures with late development degenerate first with age (Douaud et al., 2014) and that the DG develops later after birth. Indeed, its functioning is impaired early in aging (Snyder, 2019). Along with the subventricular zone, which is the only region with the ability to generate adult‐born new neurons, the DG plays a key role in increased episodic memory functions, and both processes are mainly affected during aging and neurodegenerative diseases (Kempermann et al., 1998; Kuhn et al., 1996). For these reasons, the rejuvenation of this area would be a significant milestone.

Recently, a mixture of four specific metabolites has been shown to enhance cellular plasticity, inducing limited reprogramming to a more flexible cell state (Hernandez‐Benitez et al., 2024). In this way, treatment with these metabolites accelerated repair after muscle injury in young and aged mice. Since partial reprogramming (bringing a cell to a more youthful state without dedifferentiating it) in transgenic models overexpressing YF has led to improvements in various aged tissues (Browder et al., 2022; Hishida et al., 2022; Ocampo et al., 2016), including the brain (Anton‐Fernandez, Roldan‐Lazaro et al., 2024b; Lu et al., 2020; Rodriguez‐Matellan et al., 2020), our hypothesis is that the effect found by Hernandez‐Benitez et al. (Hernandez‐Benitez et al., 2024) could to some extent replicate the partial reprogramming strategy in brain cells. To assess this objective, we evaluated the potential reversion of different age‐associated changes in the brain due to hippocampal infusion of these 4‐compound cocktail metabolites in aged wild‐type mice. In this study, the potential reversion of aging‐dependent biological changes was analyzed at three different levels: (a) chromatin component (histone and DNA) methylation, (b) transcriptome and (c) age‐dependent protein expression. Thus, alterations at those levels have resulted in functional changes, leading to cognitive enhancement.

Regarding the impact of aging on epigenetic changes, particularly the methylation of chromatin components, research has shown a uniform alteration in the methylation state of CpG islands within DNA as organism's age. Mathematical algorithms have facilitated the estimation of epigenetic age based on the methylation status of specific CpGs, providing insight into biological aging processes (Horvath & Raj, 2018). Aging is also associated with changes in specific histone marks and global DNA hypomethylation, coupled with hypermethylation of genes involved in key cellular processes (Benayoun et al., 2015; Zampieri et al., 2015).

Although hippocampal infusion of metabolites in 16‐month‐old C57BL/6 wild‐type mice did not result in a significant reduction in age acceleration according to Horvath's epigenetic clock method, we nevertheless observed significant changes in basal methylation levels at several age‐related loci following a single metabolite injection. In addition, these changes were five times greater in older mice than in younger mice, confirming the effectiveness of the anti‐aging effects of metabolites on aged mice. Most of the top age‐associated CpGs affected by metabolite treatment were related to nervous system‐specific genes with roles in neurodevelopment, brain cell differentiation, memory functioning or synaptic maintenance. Among these loci, the Zic4 transcription factor locus had the highest significance, followed by Cacnad2d. This finding is highly important because Zic4 has been recently described as a highly relevant age‐related gene whose hypermethylation has been correlated with a causal role in the decline of spatial memory in aged rats (Chiavellini et al., 2022). In our aged mice, metabolites induced hypomethylation of the Zic4 locus, restoring its methylation to younger levels and potentially impacting cognition.

To explore other types of epigenetic mechanisms affecting metabolites, we tested histone methylation. This is another key epigenetic mechanism involved in aging that has been found to couple with DNAm to act in concert to silence genes (Chiavellini et al., 2022; Henckel et al., 2009; Karimi et al., 2011; Ocampo et al., 2016; Qin et al., 2015; Weinberg et al., 2019). In fact, the heritable process of chromatin repression is frequently marked by the interplay of DNAm and specific histone tail methylations, such as H3K9me3 and H4K20me3 (Fuks, 2005; Henckel et al., 2009; Stancheva, 2005), both of which are epigenetic marks that are strongly implicated in the maintenance of heterochromatin and in the aging process (Benayoun et al., 2015; Liu, Cao, et al., 2013; Zhang et al., 2015). In line with this, we have shown that metabolites were able to decrease H4K20me3 levels, which have been found to be increased in the livers and kidneys of aged rats and in human fibroblasts (Benayoun et al., 2015). Since H4K20 is involved in spatial learning and memory (Wang et al., 2015), potential cognitive benefits were expected after restoring H4K20 levels. On the other hand, one‐dose treatment with metabolites at ~12 months of age was not sufficient to increase the levels of H3K9me3 in the DG. It is possible, however, that these mice could not be old enough to have significantly decreased levels of this marker, making it difficult to detect relevant changes after a single‐dose treatment. In addition, H3K9me3 could be a more constitutive and permanent modification than H4K20me3, as H3K9me3 is less dynamically methylated and unable to change with short‐term reprogramming treatment. Therefore, based on both DNA and histone methylation data, we observed that metabolite treatment appeared to induce a more youthful epigenetic pattern in the hippocampus of aged mice.

To verify potential alterations in RNA expression induced by epigenetic rejuvenation and reprogramming due to metabolite treatment, we performed RNA‐seq analysis. The injection of metabolites in aged C57BL/6 mice induced changes in the expression of genes involved mainly in neuronal differentiation, neurodevelopment, proliferation, or posttranslational modifications, suggesting that the treatment could induce partial reprogramming of DG cells. In terms of changes in protein expression observed in the hilus and SGZ, a significant increase in the immunoreactivity of the YF Sox2 was detected, indicating potential signs of cellular reprogramming (Anton‐Fernandez, Cuadros, et al., 2024). In any case, the increase in the Sox2+/GFAP+ population, whose abundance in the DG is severely reduced with age (Alonso, 2001; Nacher et al., 2003), along with the increase in proliferation in the SGZ, which is the stage at which neurogenesis is most affected by aging (Cameron & McKay, 1999; Kempermann et al., 1998; Kuhn et al., 1996; Kuipers et al., 2015; Lemaire et al., 2006; Molofsky et al., 2006), pointed again to a global rejuvenation of the DG due to metabolite treatment.

Moreover, the enhancement of adult neurogenesis, particularly the increase in the survival of newly generated neurons (double‐labeled for doublecortin and CldU) resulting from metabolite treatment, could reverse the significant decline observed with aging in terms of the differentiation of new cells into neurons (Bizon et al., 2004; Bondolfi et al., 2004; Driscoll et al., 2006; Heine et al., 2004; Kempermann et al., 1998; Lichtenwalner et al., 2001; McDonald & Wojtowicz, 2005; Molofsky et al., 2006; Nacher et al., 2003). All these results could be explained by the involvement of 1C metabolites in the self‐renewal, proliferation, and neuronal differentiation of neural stem cells (NSCs) observed previously both in vitro and in adult hippocampal NSCs (Kruman, et al., 2005; Liu et al., 2013; Luo et al., 2013; Zhang et al., 2008).

Finally, changes in DNA or histone methylation are also involved in synaptic plasticity and memory processes. For example, methyl donor deficiency has been found to alter the DNAm of genes involved in learning and memory in neurons, resulting in diminished memory consolidation, impaired novel object recognition (NOR), and alterations in the methylation pattern of the glutamate receptor gene Gria1 (Tomizawa et al., 2015). The Gria1 protein is directly involved in synaptic plasticity and is positively regulated by genes such as MHY7B, whose expression increased with our metabolite treatment. In addition, downregulation of some isoforms of the Zic family led to a reduction in the expression of NMDA subunit genes, such as GluN2C, in the cerebellum (Frank et al., 2015). Furthermore, 6‐week‐old mice fed a methyl‐donor‐deficient diet (folate‐methionine‐choline deficient) showed fear memory acquisition impairment along with a reversible decrease in the gene expression of Grin2b, which encodes the GluN2b subunit of the NMDA receptor, a key neuronal plasticity marker (Ishii et al., 2014). Accordingly, in this study, we observed that the administration of metabolites to the DG induced an increase in the GluN2B subunit, which is predominantly expressed in young animals (Tang et al., 1999) but does not affect the expression of the GluR1 subunit, which is characteristic of mature neurons. These same results were in accordance with our previous work with Yamanaka‐overexpressing mice (Rodriguez‐Matellan et al., 2020), supporting a partial reprogramming effect of metabolites in the DG.

In line with this, our recent findings (Anton‐Fernandez, Cuadros, et al., 2024) revealed that activating the folate alpha 1 receptor in the brain has similar effects on GluN2B receptor subunits, as does increasing the expression of YF such as Sox2 or Klf4, in conjunction with cognitive enhancement. The detection of FRα1 activation subsequent to treatment with the metabolites in this study implies that, at least, FRα1 activation may constitute one of the mechanisms through which partial reprogramming in the DG of aged mice is achieved.

In summary, treatment with a single dose of four compound cocktail metabolites partially restored the DNAm patterns of several age‐associated *loci*, which are mainly associated with memory or synaptic functions. The main consequence of these molecular improvements has been the enhancement of memory, whose impairment is strongly associated with age. Therefore, the external one‐dose administration of metabolites to aged mice was enough to induce the rejuvenation of the DG epigenome and of mouse cognition. For these reasons and considering their ability to cross the blood–brain barrier, these metabolites should be considered potential targets for cognitive anti‐aging treatments in the future.

## 4 MATERIALS AND METHODS

### 4.1 Animals

A total of 32 C57BL/6 male and female mice (21 and 11, respectively) were bred in the animal facility at the Centro de Biología Molecular Severo Ochoa, housed in a specific pathogen‐free colony facility under standard laboratory conditions following European Community Guidelines (directive 86/609/EEC), and handled in accordance with European and local animal care protocols (PROEX 62/14 and 291/15). The animals were housed 4–5 per cage with food and water available ad libitum and maintained in a temperature‐controlled environment on a 12/12‐h light/dark cycle with light onset at 8 a.m.

### 4.2 Metabolites

We used the medium 1C‐MIM, which was donated by Izpisua‐Belmonte's laboratory, to perform blind experiments in brain cells. This medium was a cocktail with 4 different metabolites, methionine, threonine, putrescine, and glycine, whose characterization has been previously described (Hernandez‐Benitez et al., 2024). We avoided the use of the other 1C‐MIM cocktail composed of 6 metabolites due to the deleterious effect of SAM or cysteine on neurons found previously (Hernandez‐Benitez et al., 2024).

This medium is preregistered on the intellectual property application # US2021/024938 under processing.

### 4.3 Experimental design

To test the effects of metabolites on the DG, we first injected 13 C57BL/6 mice (~12 months old) with 1C‐MIM (5 mM) solution (# US2021/024938) or vehicle (Neurobasal+B27) in the hilus region of both brain hemispheres (Figure S3). One week after the intracerebral injections, the mice were subjected to behavioral (open field trial) and memory (novel object recognition) tests to study the potential functional effects of the metabolites on the CNS. After the final memory tests were conducted, the animals were perfused (2 weeks after surgery). To perform epigenetic methylation analysis, we needed to increase the N of the animals; the same protocol was repeated for another group of 19 C57BL/6 mice, which were divided into two groups of different ages (eight 3‐4‐month‐old and eleven 14‐16‐month‐old mice).

### 4.4 Stereotaxic surgery and intrahippocampal microinjection

After anesthesia with isoflurane, the mice were secured in a stereotaxic frame (Kopf). Holes were drilled bilaterally in the skull at the injection sites (one per hemisphere) via a microdrill with a 0.5 mm drill bit. The stereotaxic coordinates used for intrahippocampal injections were as follows (from bregma): anterior–posterior 2.2, lateral 1.4, and dorsoventral 2.0. A 33‐gauge needle Hamilton syringe, mounted to the stereotaxic frame, was used to inject 2 μL of vehicle or metabolites at each site. Injections occurred at a rate of 0.25 μL/min, after which the needle was left in place for an additional 3 min, stopping for another 3 min halfway. After the injections were completed, the skin was sutured, the mice received intradermal buprenorphine (0.1 mg/kg) injection, and the animals were allowed to recover for 1 h on a heating pad before returning to the home cage. The mice remained in the home cage for an additional week before the start of behavioral testing, during which they were given ibuprofen in their drinking water for 5 days. The mice were then sacrificed 2 weeks after surgery (see Figure S3).

### 4.5 CldU and IdU injection protocol

We injected two different thymidine analogs at different times into the same animals: 2 weeks and 24 h prior to sacrifice each one. Mice received four injections of 5‐chloro‐2′‐deoxy‐uridine (CldU i.p. 42.75 mg/kg bw) every 2 h (200 μL/injection on alternative sides of the body) 24 h before cranial surgery and microinjections of the solutions. In this way, we were able to determine the effect of treatment on the survival of new 2‐week‐old neurons. The day before perfusion, the mice were injected with 5‐iodo‐2′‐deoxyuridine (IdU, i.p., 57.65 mg/kg bw) following the same protocol used for CldU. With IdU, we could follow the effects of treatment on the proliferation of new cells.

### 4.6 Sacrifice and tissue processing

Mice were anesthetized with an IP pentobarbital injection (Dolethal, 60 mg/kg body weight) and transcardially perfused with saline. The brains were separated into two hemispheres. One hemisphere was removed and fixed in 4% paraformaldehyde.

In 0.1 M phosphate buffer (PB; pH 7.4) overnight at 4°C. The next day, the sections were washed three times with 0.1 M PB and cut along the sagittal plane using a vibratome (Leica VT2100S). Serial parasagittal sections (50 mm thick) were cryoprotected in 30% sucrose solution in PB and stored in ethylene glycol/glycerol at 20°C until analysis. In the other hemisphere, the hippocampus was rapidly dissected on ice and frozen in liquid nitrogen for later use in RNA‐seq and DNAm analysis.

### 4.7 Epigenetic clock

After euthanasia, the tissues were quickly extracted and flash‐frozen in liquid nitrogen. Samples were minced on a liquid nitrogen‐resistant mortar, and then samples from 25 to 50 mg of ground frozen tissue were separated for genomic DNA extraction. DNA was processed by the Clock Foundation (https://clockfoundation.org/data‐tools/), which retrieved the averaged results of biological replicates per condition (Moqri et al., 2023).

### 4.8 RNAseq analysis

RNA was isolated in a Maxwell 16 Instrument using a Maxwell 16 LEV simple RNA tissue kit (Promega, P.N. AS1280) according to the manufacturer's instructions (Promega, P.N. AS1280). The sample concentration, purity, and integrity of the RNA were quantified with a Nanodrop One spectrophotometer (Thermo Fisher Scientific). The RNA integrity of the samples was also checked with an Agilent 2100 Bioanalyzer.

The original RNAseq data included 1 × 13 read sets in FASTQ format, which were sequenced in two runs, and quality analyses were performed on the reads using FastQC1 software. The reference genome and the annotation file of *M. musculus* (mm10) were downloaded from UCSC ftp site2 and GENECODE3, respectively. The reads were aligned against the *M. musculus* genome using Hisat24 aligner, a fast and sensitive alignment program for mapping next‐generation sequencing reads to a reference genome.

We used HTSeq‐count7 to count the reads mapping each feature. In an RNA‐seq experiment, these features correspond to genes, where each gene is considered to be the union of all its exons. We used the “intersection‐strict” resolution mode, where reads are counted only if they are inside a gene or inside the exons of a gene. If a read was positioned in more than one gene, only the first one was considered. Differential expression analysis was performed using DESeq28, an R software package. This software calculates gene and transcript expression levels in more than one condition and tests them for significant differences using the negative binomial distribution, which estimates variance–mean dependence in RNA‐seq count data. It is more sensitive and precise than other methods, maintaining control over the false positive rate. Heatmap and hierarchical clustering of DEGs were performed using the heatmap function in the stats package in R. For data analysis, we received support from the Genomics and NGS Core Facility at the Centro de Biología Molecular Severo Ochoa (CBM, CSIC‐UAM), which is part of the CEI UAM + CSIC, Madrid, Spain.

### 4.9 Immunohistochemistry techniques

Before staining, the sections were washed with phosphate‐buffered saline (PBS) to eliminate the cryoprotective buffer and immersed in 1% H_2_O_2_ in PBS for 45 min to quench endogenous peroxidase activity. The sections were immersed for 1 h in blocking solution (PBS containing 0.5% fetal bovine serum, 0.02% Triton X‐100, and 1% bovine serum albumin) and incubated overnight at 4°C with Glun2B primary antibody (Y1336, PhosphoSolutions) or caspase‐3 (D175, Cell Signaling Technology) diluted in blocking solution. After washing, the brain sections were incubated first with a biotinylated anti‐rabbit secondary antibody and then with an avidin‐biotin complex using an Elite Vectastain kit (Vector Laboratories, PK‐6101‐2). Chromogen reactions were performed with diaminobenzidine (SIGMAFASTTM DAB; Sigma, D4293) for 10 min. Hippocampal sections were coverslipped with FluoroSave. Images were captured using an Olympus BX41 microscope with an Olympus Microscope Digital Camera Model DP71 (Olympus Denmark).

### 4.10 Immunofluorescence techniques

For immunofluorescence experiments, free‐floating serial sections (50 μm thick) were first rinsed in PB and then preincubated for 2 h in PB supplemented with 0.25% Triton‐X100 and 3% normal serum from the species in which the secondary antibodies were raised (normal goat serum/normal donkey serum, Invitrogen, Thermo Scientific). For the study of proliferation and neurogenesis, serial sections were previously pretreated in 2 M HCl for 15 min at 30°C. Subsequently, brain sections were incubated for 24 h at 4°C in the same preincubation stock solution containing the following primary antibodies in different combinations: D175 for caspase‐3 (Cell Signaling Technology); ab6326 (Abcam) for CldU; AB2253 (Sigma Aldrich) for doublecortin; IF03L (Millipore) for GFAP; AB31232 (ABCAM) for GluR1; ab8898 (Abcam) for histone H3 (trimethyl K9); ab78517 (Abcam) for histone H4 (dimethyl K20, trimethyl K20); 347,580 (BD Biosciences) for IdU; p1516‐1480 (Phosphosolution) for the NMDA NR2B subunit; 07–750 (Millipore) for phosphohistone H3 (Ser10); and AF2018 (RD SYSTEM) for Sox2.

After rinsing in PB, the sections were incubated for 2 h at room temperature with the appropriate combinations of Alexa 488–594–647 conjugated donkey/goat anti‐mouse/rabbit/guinea pig or rat secondary antibodies (1:500; Molecular Probes, Eugene, OR, USA). Sections were also stained with the nuclear stain 4′,6‐diamidino‐2‐phenylindole (DAPI; Sigma, St. Louis, MO, U.S.A.).

From the whole DG of the hippocampus, we obtained stitched image stacks recorded at 1.5 μm intervals through separate channels with a 20x lens (Nikon A1R confocal microscope, NA 0.75, refraction index 1, image resolution: 1024 × 1024 pixels). Adobe Photoshop (CS4) software was used to construct the figures.

### 4.11 Immunohistochemistry quantification

The density of caspase‐3‐, DCX‐, Sox2‐, CldU‐, IdU‐ and PH3‐positive cells in three/four different close brain sections was quantified by counting the number of cells localized in each whole DG, distinguishing between the SGZ and GCL, and the adjacent hilus region. In all cases, the sections were always closer to the injection area and then localized at the same positions. For determination of the density of different cells, the DG was traced on the DAPI channel of the z projection of each confocal stack of images, and by using the freehand drawing tool in Fiji, the area of this structure was measured. This area was multiplied by the stack thickness to calculate the reference volume. The number of positive cells was divided by the reference volume, and the density (number of cells/mm^3^) of the cells was calculated.

For H3K9me3 and H4K20me3 antibodies, the acquisition confocal settings (laser intensity, gain, pinhole) were kept constant for all images, which were captured in the same confocal session. Using the DAPI channel, the whole GCL area was previously selected as an ROI. An invariant subtract background and threshold was then set in Fiji for both channels. The area above the threshold was then measured in Fiji.

For GluN2B and GluR1 antibodies, quantification was carried out by measuring the stained area with the ImageJ program. The whole area occupied by each subunit marker in granular cell dendrites situated in the molecular layer of the DG was measured using the color deconvolution function of ImageJ and the H‐DAB vector.

### 4.12 Nuclear fractionation of cultured human cells and western blotting

The presence of FRα in the nuclear fraction and the expression of Klf4 were demonstrated by cellular fractionation followed by Western blot analysis. SK‐N‐SH human neuroblastoma cells were treated with metabolites (0.5 mM, 1 mM) or vehicle solution (neurobasal medium supplemented with B27) for 30 min at 37°C. Adherent cells were rinsed with ice‐cold PBS and then scraped into ice‐cold hypotonic Buffer A (20 mM HEPES pH 7, 0.15 mM EDTA, 0.015 mM EGTA, 10 mM KCl, 1% NP‐40 supplemented with protease inhibitors). Following a 30‐minute incubation on ice with gentle agitation, the cells were pelleted by centrifugation at 2300 rpm for 5 min at 4°C. The resulting supernatant was collected as the cytosolic fraction. The nuclear pellet was then washed in five volumes of buffer B (10 mM HEPES pH 8, 25% (v/v) glycerol, 0.1 M NaCl, and 0.15 mM EDTA). Following centrifugation, the nuclei in the pellet were resuspended in two volumes of Buffer A.

Whole‐cell lysates or cell nuclear pellets from each experiment were then quantified by the BCA protein assay. The samples were separated by 10% SDS–PAGE and electrophoretically transferred to a nitrocellulose membrane (Schleicher & Schuell GmbH). The membrane was blocked by incubation with 5% semi fat dried milk in PBS and 0.1% Tween 20 (PBS), followed by a 1‐h incubation at room temperature with the primary antibody in PBS. The following primary antibodies were used: anti‐FRα 1 (1/300; Thermo Fisher, ref: PA5‐101588) for the nuclear fraction, anti‐Krüppel‐like factor 4 (Klf4, 1/500; R&D system, ref: af3158) for the cytosolic fraction, and Lamin B1 (1/1000; Santa Cruz SC‐377000) and β‐actin (1/10000; SIGMA, ref: A5441) as the respective loading controls. After three washes, the membrane was incubated with a horseradish peroxidase‐conjugated anti‐rabbit and mouse Ig (DAKO) conjugate, followed by several washes in PBS‐Tween 20. The membrane was then incubated for 1 min in Western Lightning reagents (PerkinElmer Life Sciences). Blots were quantified using an EPSON Perfection 1660 scanner and ImageJ software.

### 4.13 Open Field, NOR, and Y‐maze tests

Mice were tested as described previously (Engel et al., 2006), with a few modifications. Locomotor activity as well as anxiety and depression‐like behaviors in old mice were evaluated using an open field test. This test involved habituation to the NOR test on the first day. In brief, the mice were habituated for 10 min in a 45 × 45‐cm plastic box with vertically opaque walls. On the second day, they were placed in the same box for 5 min, after which they were allowed to explore two identical objects (objects A and B). Both objects were placed on the long axis of the cage, each 13 cm from the cage end. After each exposure, the objects and the cage were wiped with 70% ethanol to eliminate odors. Two hours after the familiarization trial, each mouse was released into the open field with one of the old objects (object A) or a new object (object C). The position of object C was the same as that of object B in the familiarization trial. The mice were given 5 min to explore the box (Test 1). Five days later, the mice were released again into the open field, with object A in the same position and another new object (object D). The position of the latter was the same as that of object C in Test 1. The animals were allowed 5 min to explore the box (Test 2). The animals were considered to show recognition when their head was <2 cm from the object. In Test 1, the time (tA and tC) the animal spent exploring the two objects (objects A and C, respectively) was recorded. The MI, defined as the ratio of time spent exploring the new object to the time spent exploring both objects (MI = [tC/(tA + tC)] × 100), was used to measure nonspatial memory. In Test 2, the same MI was applied, replacing tC with the time spent exploring object D, tD (MI = [tD/(tA + tD)] × 100).

To evaluate spatial memory, a Y‐maze test was conducted. This test relies on the natural curiosity of rodents to explore new environments. Mice were initially placed in one of the arms of a black Y‐maze apparatus, which consists of three plastic arms forming a “Y” shape. They were allowed to explore the maze for 10 min, with one arm closed off (training trial). After a one‐hour interval, the mice were returned to the Y‐maze in the same start arm and given 5 min to explore all arms. The time each mouse spent in each arm was recorded via video and analyzed using AnyMaze software. Spatial memory performance was measured by the MI, defined as the ratio of time spent exploring the previously closed arm to the time spent exploring both the closed and open arms.

### 4.14 Statistical analysis for immunohistochemical and behavioral assessments

The data are presented as the mean values ± SEMs. Statistical analyses were performed using GraphPad Prism 8. The experiments, as well as the acquisition and analysis of all the data, were conducted in a randomized order by investigators who were blinded to the experimental conditions. To compare the two experimental groups (mice injected with vehicle or metabolites), the data were previously tested for normality using the Shapiro–Wilk test, and an unpaired Student's *t*‐test was carried out. To study the behavior of young and aged mice treated with vehicle or metabolites, a two‐way ANOVA was performed with Fisher's LSD post hoc test. For WB analysis, a Kruskal–Wallis test followed by Dunn's multiple comparison test was performed. A 95% confidence interval was applied for statistical comparisons.

## AUTHOR CONTRIBUTIONS

AAF designed and carried out the experiments and wrote the manuscript; RPC conducted some experiments; and FH and JA conceptualized and wrote the manuscript.

## FUNDING INFORMATION

Work in the laboratory of JA is funded by grants from the Spanish Ministry of Economy and Competitiveness (PGC‐2018–09177‐B‐100) and PID2021‐123859OB‐100 from MCIN/AEI/10.13039/501100011033/FEDER, UE. This work was also supported by the CSIC through an intramural grant (201920E104). Work in the laboratory of FH is funded by grants from the Spanish Ministry of Economy and Competitiveness (Ministerio de Economía, Industria y Competitividad, Gobierno de España, PID2020‐113204GB‐I00) and was cofinanced from the Comunidad de Madrid through Structural Funds of the European Union [S2017/BMD‐3700 (NEUROMETAB‐CM)]. The Centro de Biología Molecular Severo Ochoa (CBM) is a Severo Ochoa Center of Excellence (MICIN, award CEX2021‐001154‐S).

## CONFLICT OF INTEREST STATEMENT

The authors declare that they have no competing interests to disclose.

## Supporting information


Figure S1.

## Data Availability

The datasets generated during the current study are available from the corresponding author upon reasonable request. The raw RNA‐seq data in this study are available in the European Nucleotide Archive under the ENA accession number PRJEB44560.
