# Supplementary material for: Hippocampal rejuvenation by a single intracerebral injection of one‐carbon metabolites in C57BL6 old wild‐type mice
Source: Aging Cell. 2024 Oct 8;24(1):e14365. doi: 10.1111/acel.14365 (PMC11709095; doi:10.1111/acel.14365)
Supplement: Supplementary file 1 — Figure S1. [file ACEL-24-e14365-s001.docx]

**SUPPORTING INFORMATION**


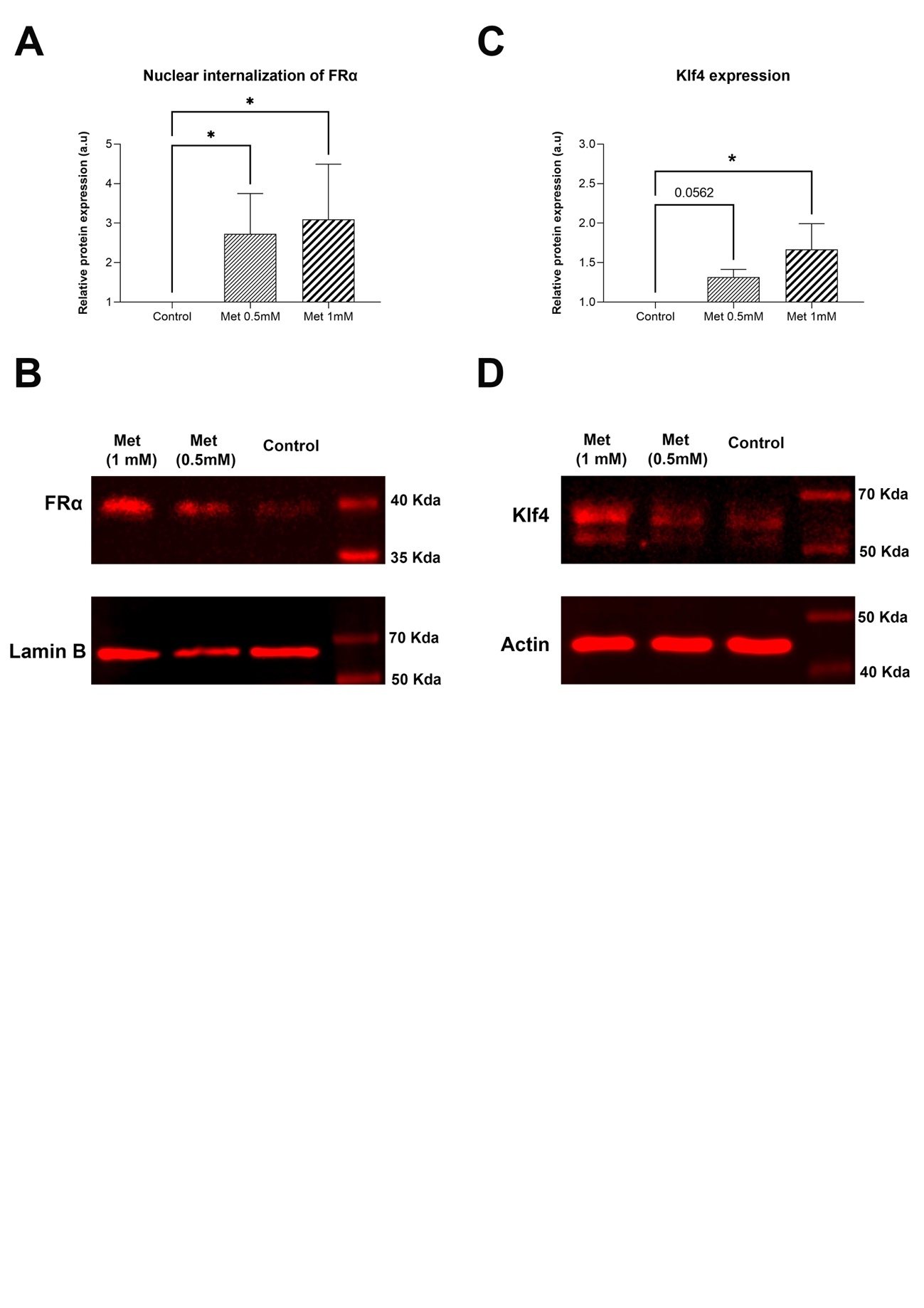


**Supplementary Figure 1. Activation of FRα1 pathway.**

A) We studied the potential activation and consequent internalization of FRα into the nucleus after using two concentrations of metabolite cocktail (0.5 and 1 mM) on SK-N-SH human neuroblastoma cells. Our results show the presence of a ~38 Kd band in the nuclear fraction in the presence or absence of metabolites. Shown in (B) are the relative protein expression level of FRα under 0.5mM and 1mM metabolites treatment in comparison to the control group, following a 30-minute incubation period. Lamin-β protein expression has been used as loading control to calculate relative protein expression of FRα. C) The addition of metabolites cocktail increases the expression of Klf4 protein in SK-N-SH cells. Shown in (D) are the relative protein expression level of Klf4 under 0.5mM and 1mM metabolites in comparison to the control group, following a 30-minute incubation period. β-actin protein expression has been used as loading control to calculate relative protein expression of Klf4 in cytosolic fraction. (Mean ± SEM; *p<0.05; **p<0.001. Kruskal Wallis with Dunn´s multiple comparisons test; (KW=7.812; p=0.0087) and (KW=7.652; p=0.0133), respectively in b and d).

**
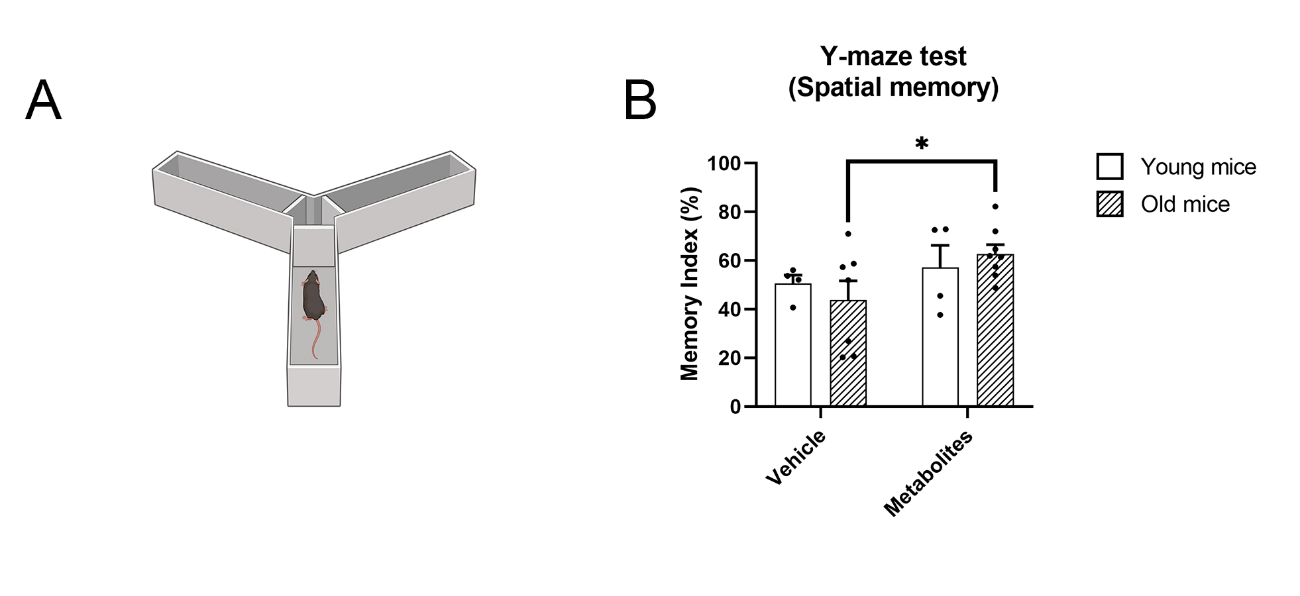
**

**Supplementary Figure 2. Spatial memory performance in young and old treated mice. A)** Representative diagram of the Y-maze apparatus. B) Bar graphs showing the results from the spatial memory test in the Y-maze. Two hours after the training phase, aged mice injected with metabolites performed significantly better than aged mice injected with the vehicle. The metabolites improved the memory index of the aged mice to levels comparable to those of the young mice. No significant differences were found between the young vehicle-infused mice and the aged mice injected with metabolites. In all histograms, black dots represent the value for each individual mouse. White bars represent the average value for young mice, while striped bars represent the average value for aged mice. The left side shows vehicle-infused mice, and the right side shows mice treated with metabolites. All data are expressed as mean ± SEM and were analyzed using a two-way Anova with uncorrected Fisher LSD post hoc test. *p<0.05.


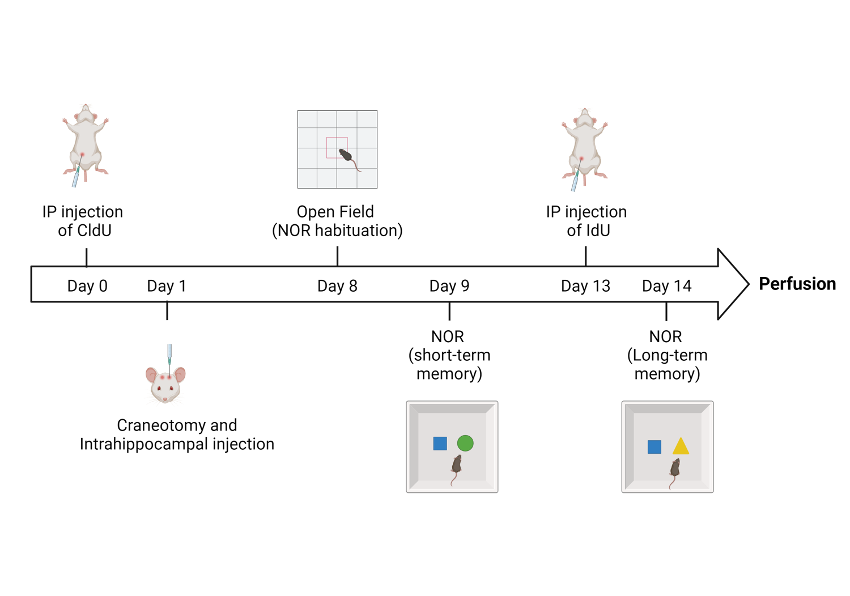


**Supplementary Figure 3. Timeline of the experiment.**
Temporal outline of the various steps performed for each mouse used in this study. The day before intracranial surgery, when the vehicle solution or metabolite cocktail was injected, the CldU thymidine analog was administered intraperitoneally. One week after surgery, the mice began behavioral tests, starting with the open field test, followed by the novel object recognition test at 2 hours and 5 days after the initial familiarization phase with the objects. The day before perfusion, another thymidine analog (IdU) was also administered intraperitoneally to the mice.
